# Supplementary material for: A positive feedback loop bi-stably activates fibroblasts
Source: Nat Commun. 2018 Aug 1;9:3016. doi: 10.1038/s41467-018-05274-6 (PMC6070563; doi:10.1038/s41467-018-05274-6)
Supplement: Supplementary file 1 — Supplementary Information [file 41467_2018_5274_MOESM1_ESM.pdf]

Supplementary Information

# The positive feedback loop formed by Twist1, Prrx1, and Tenascin-C bi-stably activates fibroblasts

Yeo, et.al.

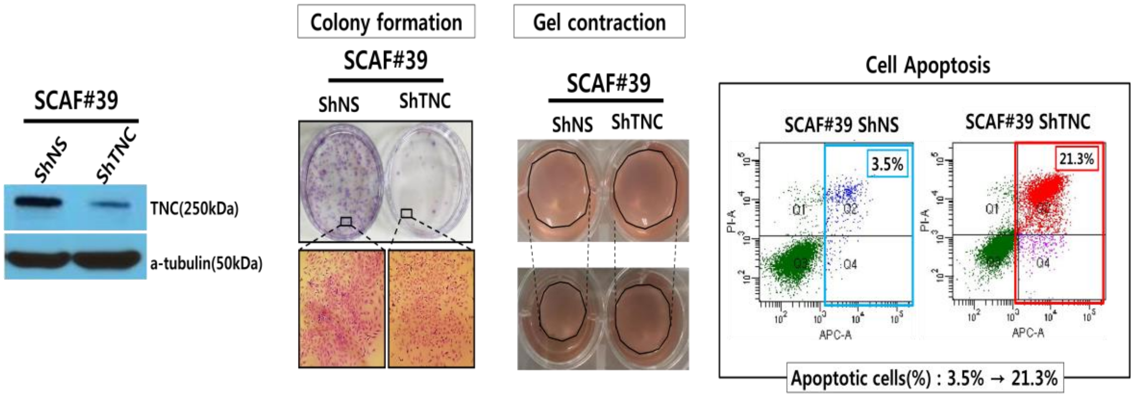


**Supplementary Figure 1.** Twist1 was co-expressed with TNC in cancer-associated fibroblast. shRNA-mediated depletion of TNC led to decreased colony formation and gel contraction abilities in stomach cancer-derived CAF (SCAF#39). Silencing of TNC also increased the proportion of apoptotic cells.

| Species | |
|---|---|
| ***Human*** | CAAAAAGTAAAGTGAGAATCCTGCTCTCTAATA***CATCTG***CAAGACATCACCCTCTCCTCCTGAAACTTT |
| ***Chimpanzee*** | CAAAAAGTAAAGTGAGAATCCTGCTCTCTAATA***CATCTG***CAAGACATCACCCTCTCCTCCTGAAACTTT |
| ***Cow*** | CGAAAAGTAAAGTGAGACTCCTGCTCTCCAATA***CATCTG***CAAGACATCACCCTCTCCTCCTGAAACTTT |
| ***Dog*** | CGAAAAGTAAAGTGAGAATCCTGCTCTCCAATA***CATCTG***CAAGACATCACCCTCTCCTCCTGAAACTTT |
| ***Gorilla*** | CAAAAAGTAAAGTGAGAATCCTGCTCTCTAATA***CATCTG***CAAGACATCACCCTCTCCTCCTGAAACTTT |
| ***Orangutan*** | CAAAAAGTAAAGTGAGAATCCTGCTCTCTAATA***CATCTG***CAAGACATCACCCTCTCCTCCTGAAACTTT |
| ***Rabbit*** | CGAAAAGTAAAGTGAGAATCCTGCTCTCTAATA***CATCTG***CAAGACATCACCCTCTCCTCCTGAAACTTT |
| ***Mouse*** | CGAAGAGTAAAGTGAGAATCCTGCTCTCTAATA***CATCTG***CAAGACATCACTCTC ----- CTGAAATTCT |
| ***Pig*** | GGAAAAGTAAAGTGAGAATCCTGCTCTCCAATA***CATCTG***CAAGACATCACCCTCTCCTCCTGAAACTTT |
| ***Horse*** | CGAAAAGTAAAGTGAGAATCCTGCTCTCCAGTA***CATCTG***CAAGACATCACCCTCTCCTCCTGAAACTTT |
| ***Cat*** | CGAAAAGTAAAGTGAGAATCCTGCTCTCCAATA***CATCTG***CAAGACATCACCCTCTCCTCCTGAAACTTT |
| ***Dolphin*** | CAAAAAGTAAAGTGAGAATCCTGCTCTCTAATA***CATCTG***CAAGACATCACCCTCTCCTCCTGAAACTTT |

**Supplementary Figure 2.** Conserved motifs in the Prrx1 promoter.
Alignment of an E-box sequence CATCTG (in bold) for 12 species.

a

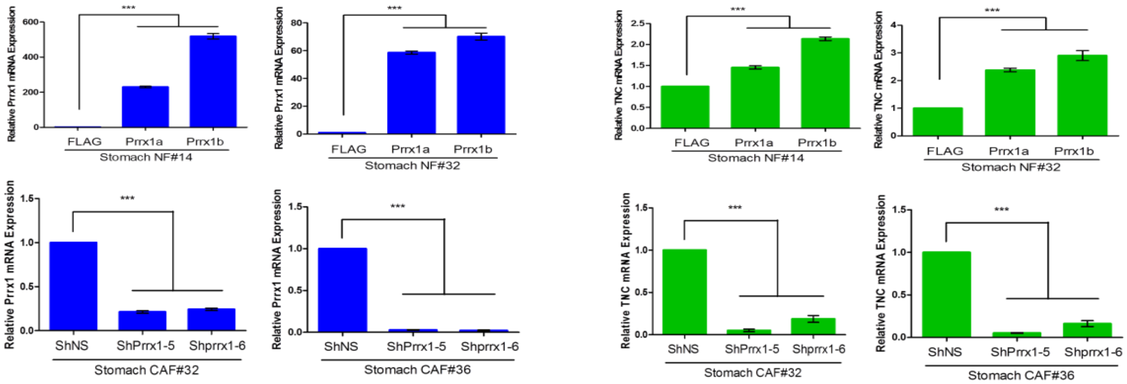


b

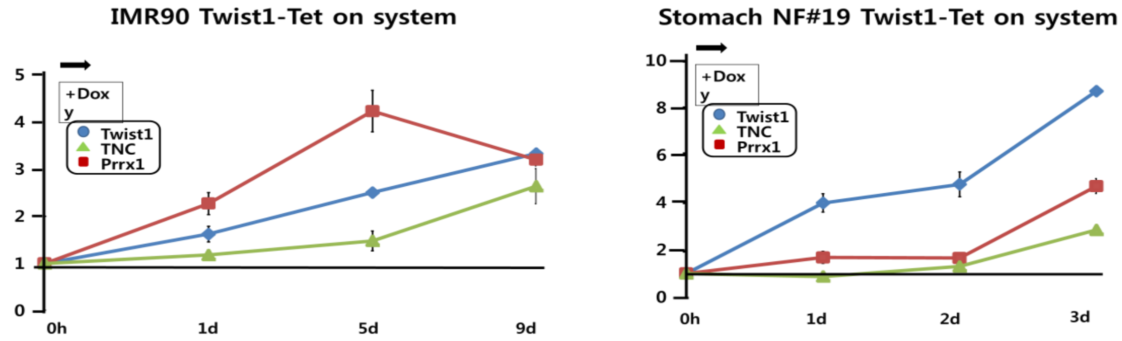


**Supplementary Figure 3.** Twist1, Prrx1, and TNC formed a positive feedback loop in fibroblasts. (a) Both Prrx1a and Prrx1b increased the mRNA level of TNC in normal fibroblasts (SNF#14, 32). The depletion of Prrx1 led to the down-regulation of mRNA levels of TNC in CAFs (SCAF#32, SCAF#39). Data are mean ± S.E.M;N=3 independent experiments.(P values, two-tailed t-test, ***p<0.001). Statistic source data for b and d are provided in Supplementary Table4. (b) Induction of Twist1 expression using Tet-On system led to sequential increases of Prrx1 and TNC mRNA expression in normal lung fibroblast (IMR#90), and normal gastric fibroblast(SNF#19)

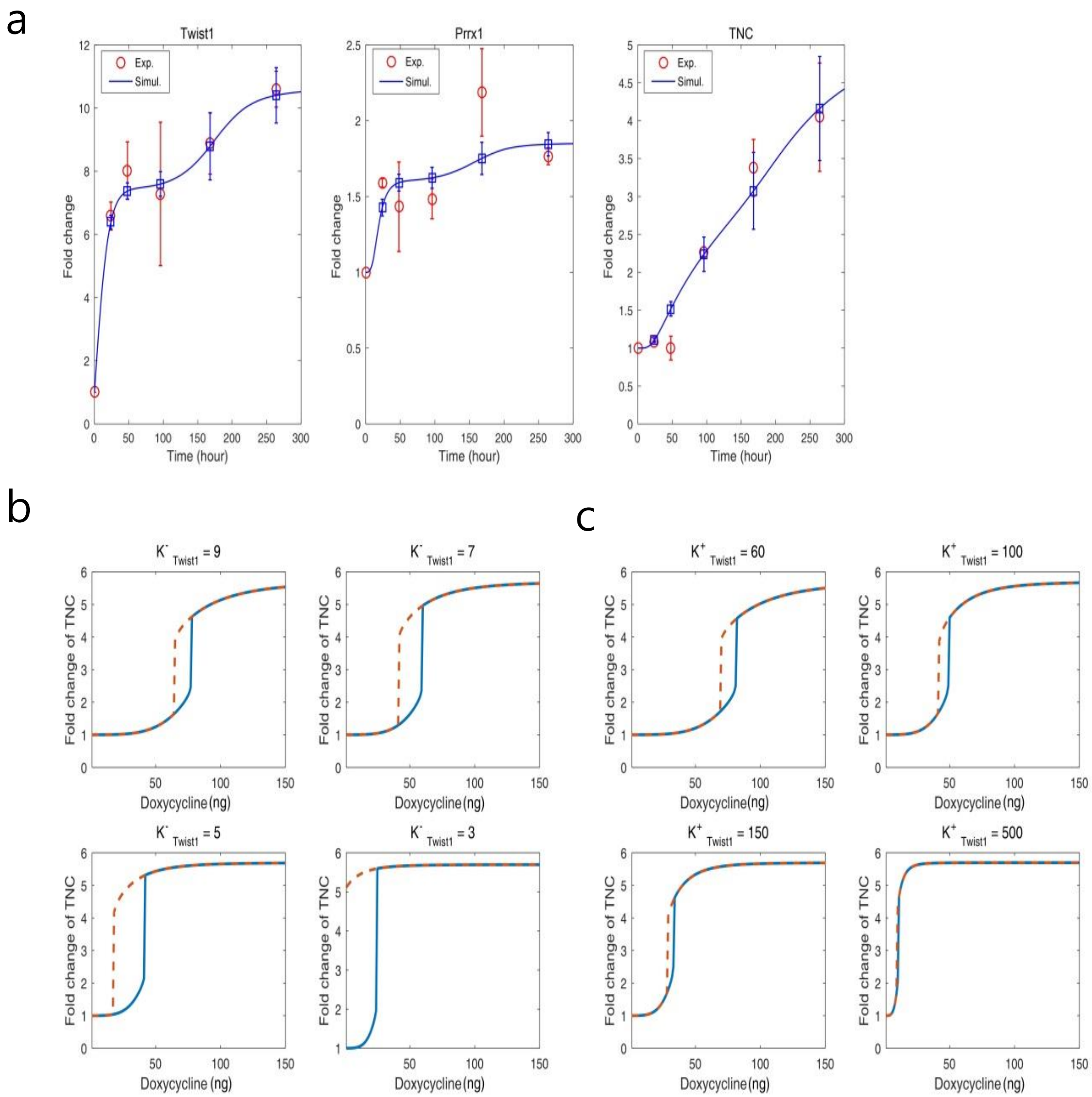


**Supplementary Figure 4.** (a)Estimation of kinetic parameter values of the mathematical model, based on temporal measurements of the fold change of Twist1, Prrx1, and TNC, for the doxycycline stimulus (100ng). Discrete data marked with circles, indicating experimental measurements of the corresponding genes (n=4, error bars indicate s.d.). The kinetic parameter values of the model were fitted to these experimental measurements. We confirmed that the simulation results are well in accord with the experimental data for 10% random perturbations of all the nominal parameter values (n=1000, error bars indicate s.d.).(b) The hysteretic responses of TNC to the doxycycline stimulus as the degradation rate of Twist1 decreases. The hysteretic curve shifts to the left along with the increase of the stability of Twist1, which leads to an irreversible state. (C)The hysteretic responses of TNC to the doxycycline stimulus, along with the increase of the production rate of Twist1. As the expression level of Twist1 increases, the hysteretic curve shifts to the left and the hysteresis gets diminished.

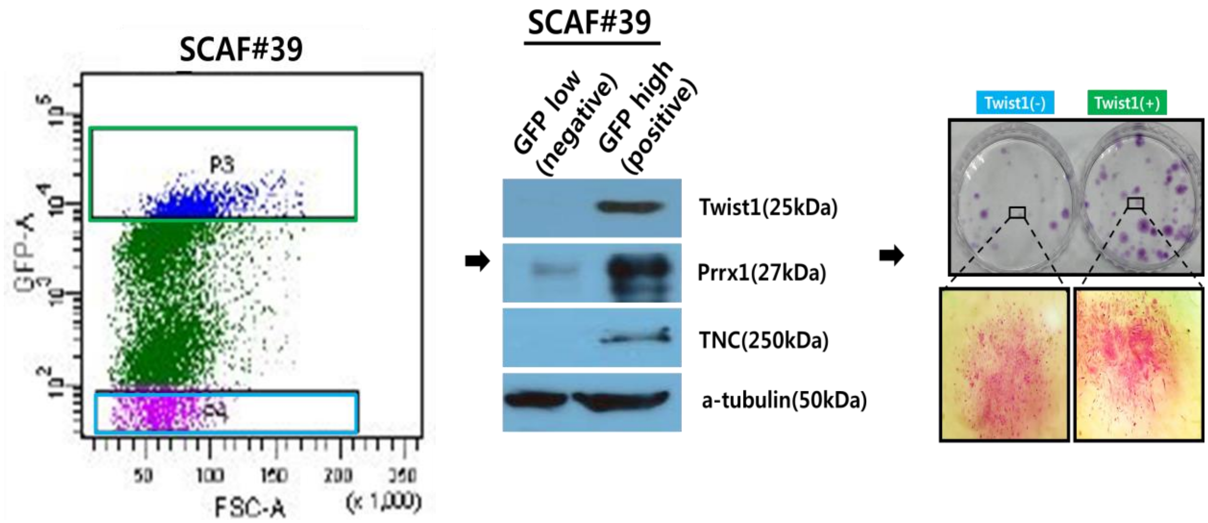


**Supplementary Figure 5.** Gastric cancer derived CAFs (SCAF#39) were sorted into Twist1-positive and negative CAFs. Twist1-positive CAFs were specifically positive for Prrx1 and TNC. Twist1-positive CAFs also exhibited increased colony forming abilities

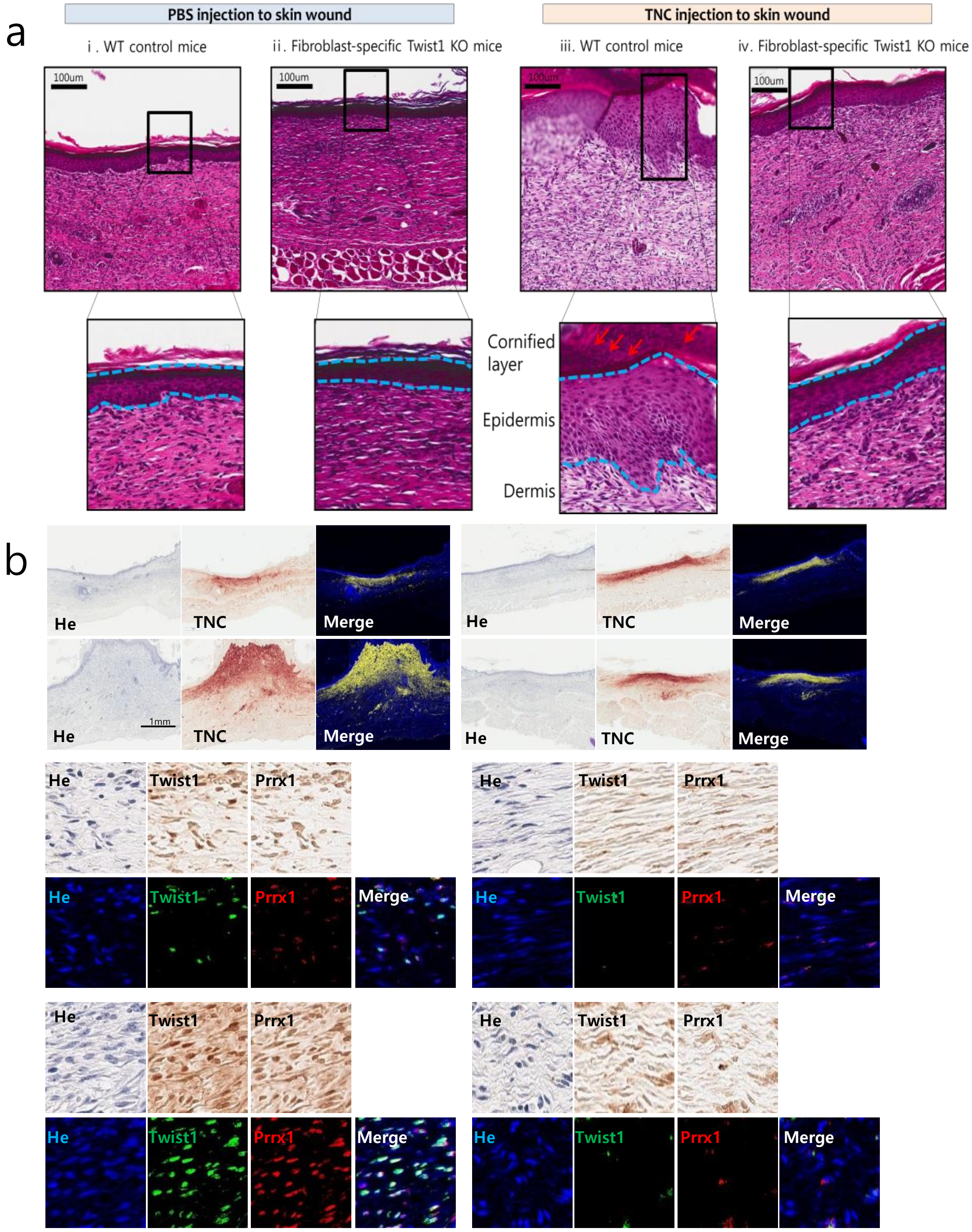


**Supplementary Figure 6**. Hyper-activation of fibroblasts and epithelial cells.
(a)TNC accumulation induced hyperplasia of both fibroblasts and epithelial cells. Acanthosis(Epithelial cells hyperplasia, blue dotted line), hyperkeratosis (a thick cornified layer), parakeratosis (presence of the nuclei in the cornified layer (red arrow)).
(b)Original IHC and multiple staining images of Fig6C,D and E.TNC-yellow, Twist1-green, Prrx1-red, He(Hematoxylin)-blue.

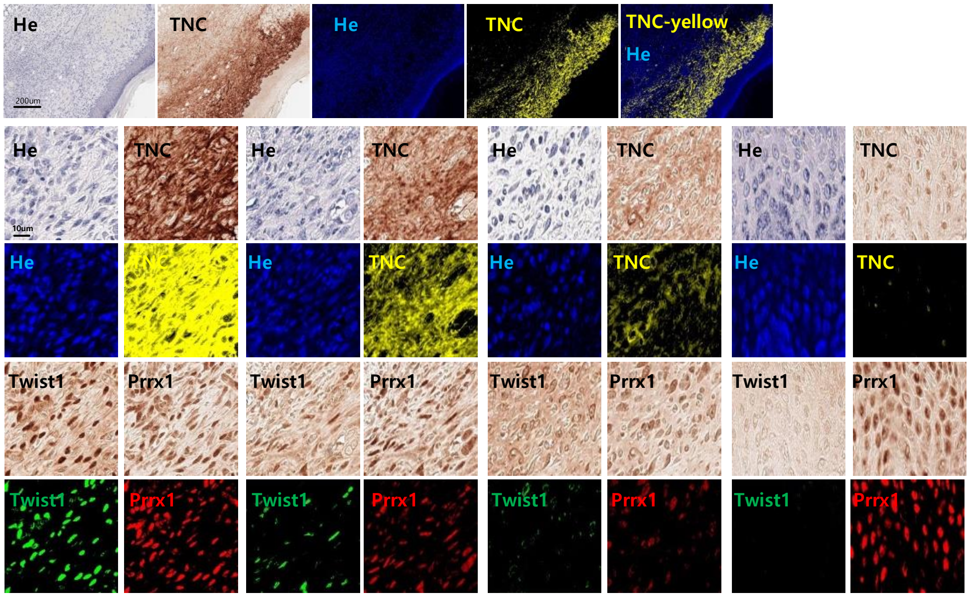


**Supplementary Figure 7**. Multiplex imaging analysis.
Original IHC and multiple staining images of Fig6C,D and E.TNC-yellow, Twist1-green, Prrx1-red, He(Hematoxylin)-blue.

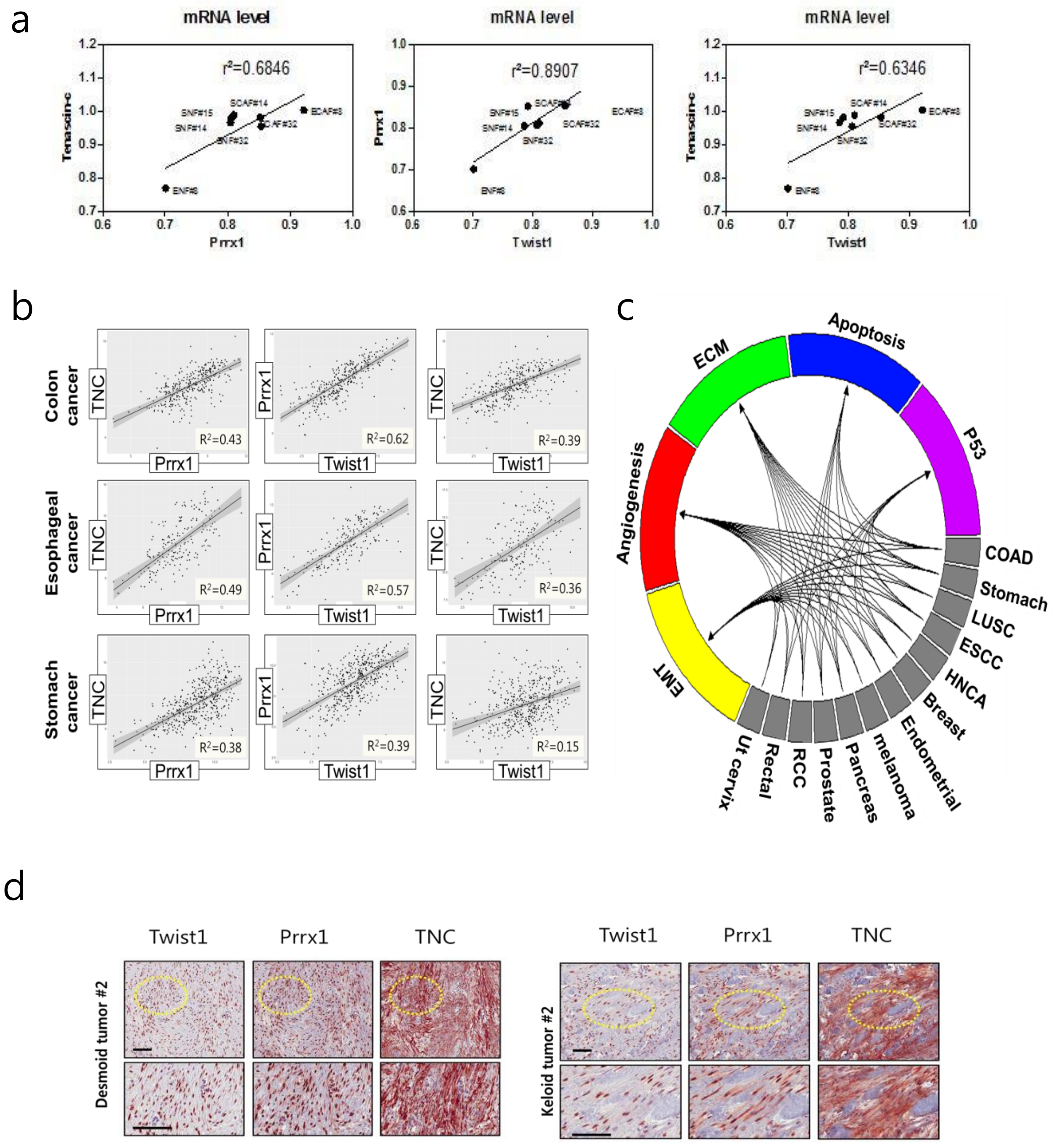
a
mRNA level
r²=0.6846
Tenascin-c
Prrx1
ECAF#8
SCAF#14
SNF#15
SNF#14
SCAF#32
SNF#32
ENF#8
mRNA level
r²=0.8907
Prrx1
Twist1
mRNA level
r²=0.6346
Tenascin-c
Twist1
b
Colon cancer
Esophageal cancer
Stomach cancer
TNC
Prrx1
Twist1
R²=0.43
R²=0.62
R²=0.39
R²=0.49
R²=0.57
R²=0.36
R²=0.38
R²=0.39
R²=0.15
c
Apoptosis
ECM
P53
Angiogenesis
EMT
COAD
Stomach
LUSC
ESCC
HNCA
Breast
Endometrial
melanoma
Pancreas
Prostate
RCC
Rectal
Ut cervix
d
Twist1
Prrx1
TNC
Desmoid tumor #2
Keloid tumor #2

e

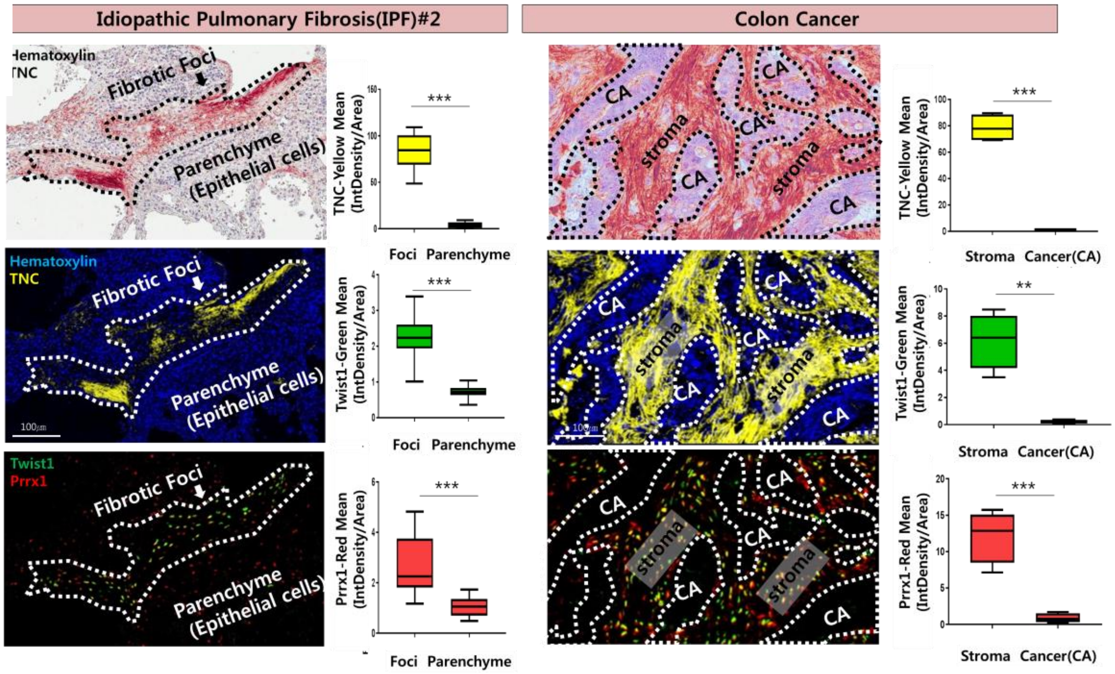

Idiopathic Pulmonary Fibrosis(IPF)#2
Colon Cancer
Hematoxylin
TNC
Fibrotic Foci
Parenchyme
(Epithelial cells)
Twist1
Prrx1
100㎛
TNC-Yellow Mean
(IntDensity/Area)
Twist1-Green Mean
(IntDensity/Area)
Prrx1-Red Mean
(IntDensity/Area)
***
**
Foci
Parenchyme
CA
stroma
Stroma
Cancer(CA)


f

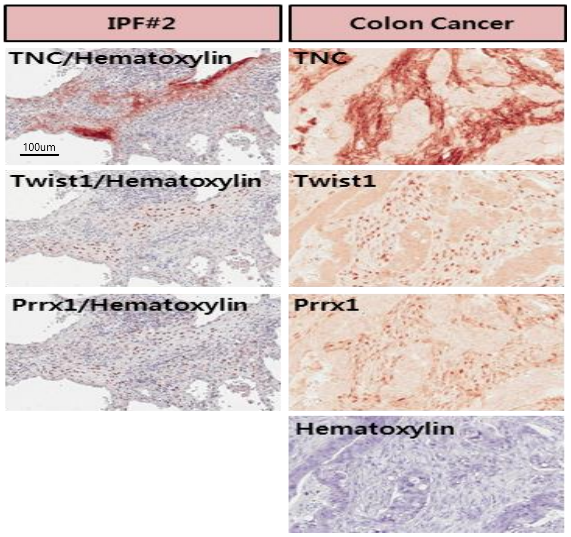

IPF#2
Colon Cancer
TNC/Hematoxylin
100um
Twist1/Hematoxylin
Prrx1/Hematoxylin
TNC
Twist1
Prrx1
Hematoxylin

**Supplementary Figure 8.** (a) Endogeneous mRNA levels of Twist1, Prrx1, TNC in various fibroblast models. (b) Analysis of RNA-SEQ data from TCGA revealed that there was a strong correlation among all three genes' expressions in various types of cancer. We downloaded the RNA-SEQ data of 1200 patients suffering from major cancer, from the homepage of The Cancer Genome Atlas (TCGA, https://cancergenome.nih.gov/). Next, we studied the correlation among three genes in a large population of cancer patients, using a public database. (c) Circos plot illustrated the results of gene set enrichment assay (GSEA). The RNA-SEQ data of 11 cancer types was obtained from TCGA. Then, Twist1/Prrx1/TNC (+/+/+) and Twist1/Prrx1/TNC (-/-/-) patients'- cohort groups were selected for each of the 11 cancer types. We then compared the gene expression patterns of both groups, and attempted to identify the specific signaling pathways enriched in the Twist1/Prrx1/TNC (+/+/+) group, by performing a gene set enrichment analysis (GSEA), using the Broad Institute's GSEA software (d) All three genes positive fibroblasts were frequently observed in pathologic fibrosis including keloid disease and desmoid tumors (aggressive fibromatosis). (e) Multiplex imaging analysis progressed from IPF#2 and Colon cancer patient tissue. Each Density/Area was measured using by image J Fiji (Twist1-green, Prrx1-red, TNC- yellow and Hematoxylin-blue). Data are mean ± S.E.M; N=3 independent measurements (P values, two-tailed t-test, *p<0.05, **p<0.001, ***p<0.0001). (f) Original IHC staining of IPF#2 and Colon cancer tissue.

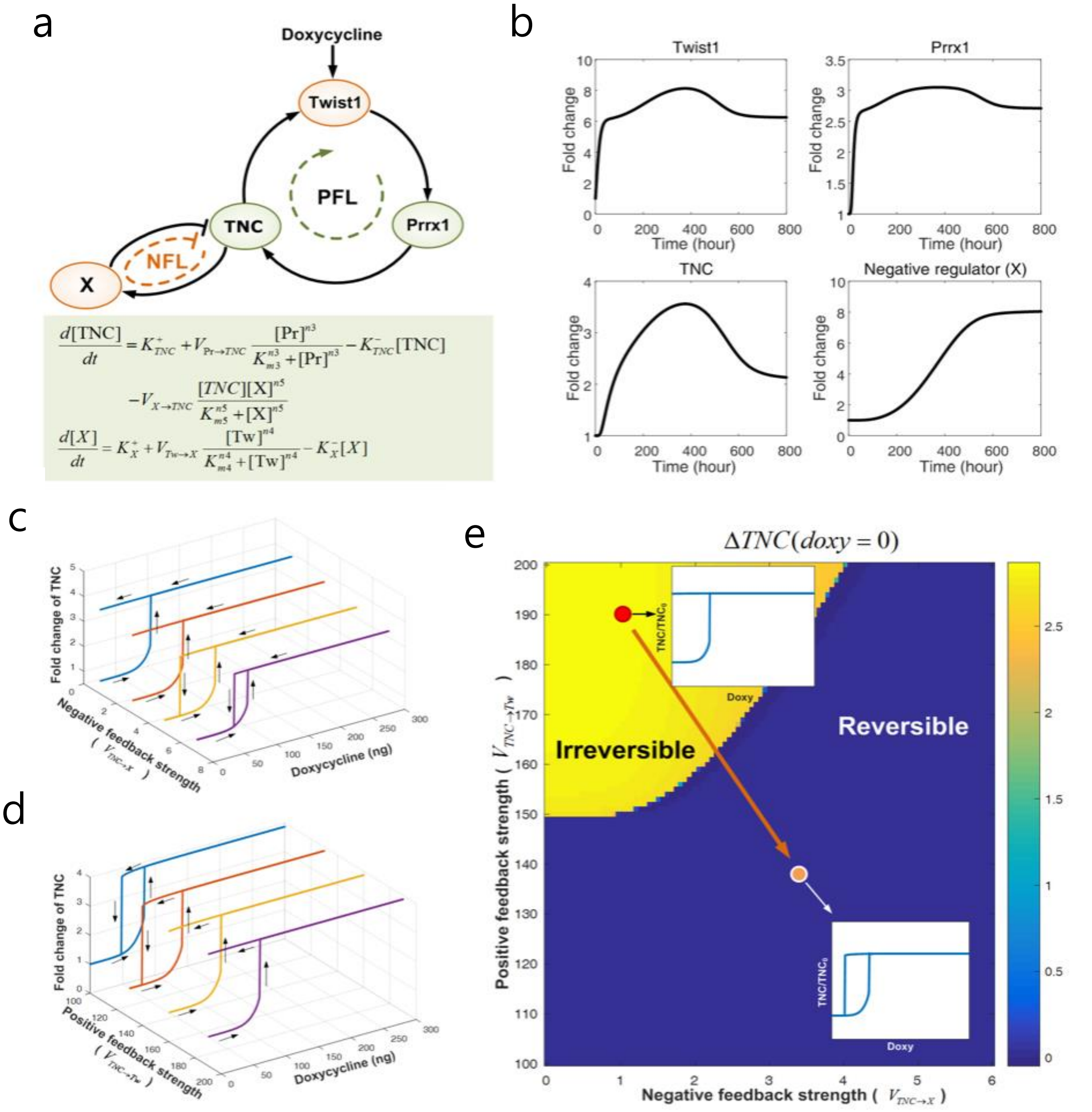


**Supplementary Figure 9**. Hysteresis analysis of a coupled positive and negative feedback loop (a) Schematic diagram of the coupled Twist1-Prrx1-TNC PFL and NFL and the mathematical description of TNC and X. The state equations for Twist1 and Prrx1 are the same as those depicted in Fig. 4(a). (b) Temporal profiles of the fold change of Twist1, Prrx1, TNC, and X for the doxycycline stimulus (100ng). (c) and (d) Various profiles for the hysteretic response of TNC to the doxycycline stimulation along with the increase of negative (c) and positive (d) feedback strength. (e) Phase diagram illustrating the irreversibility of TNC with respect to the negative and positive feedback strength (see the detailed descriptions of the irreversibility of TNC in Fig. 4(e)).

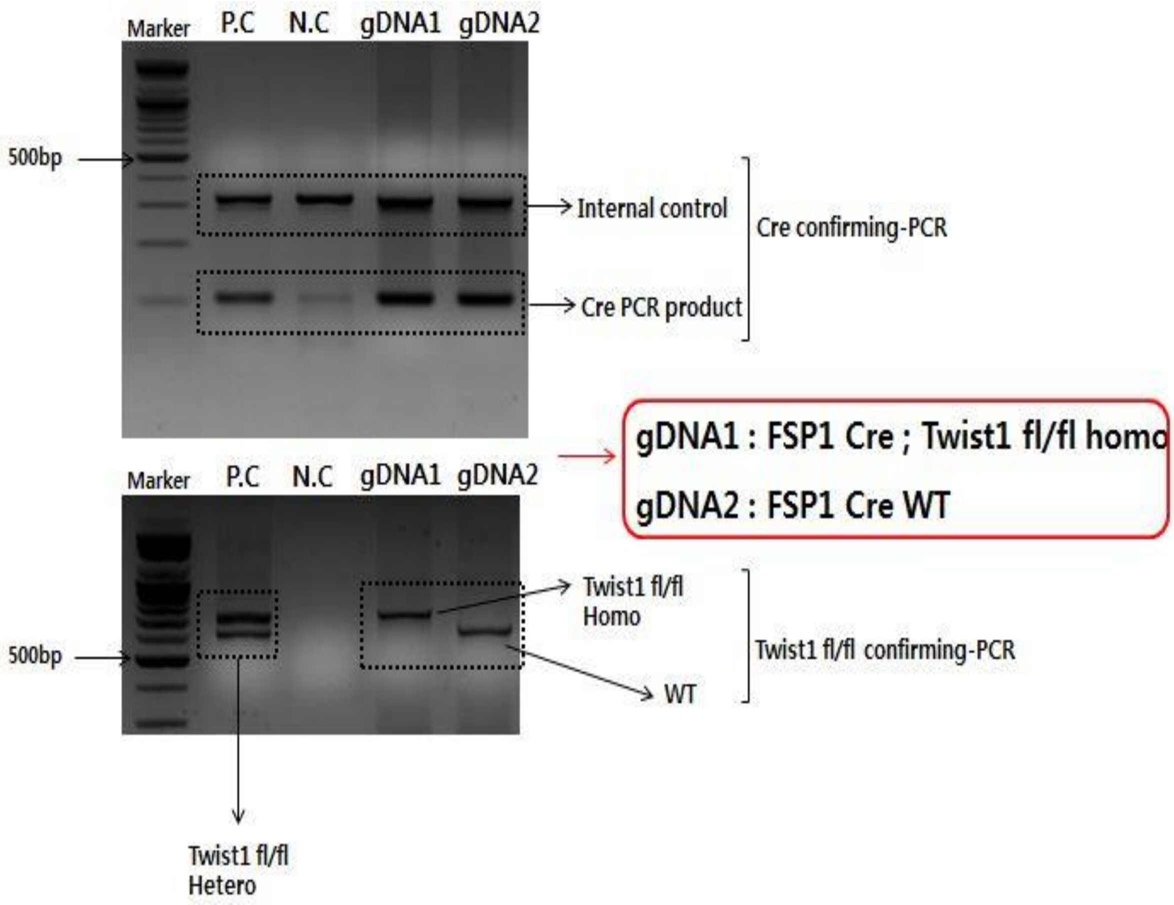


**Supplementary Figure 10**. Mice genotyping. Representative gel run image. gDNAs were isolated from each mice ear or toes. (top) confirming FSP1-Cre, (bottom) Twist1 flox/flox .

Related to Figure 1 and supplementary Figure 1

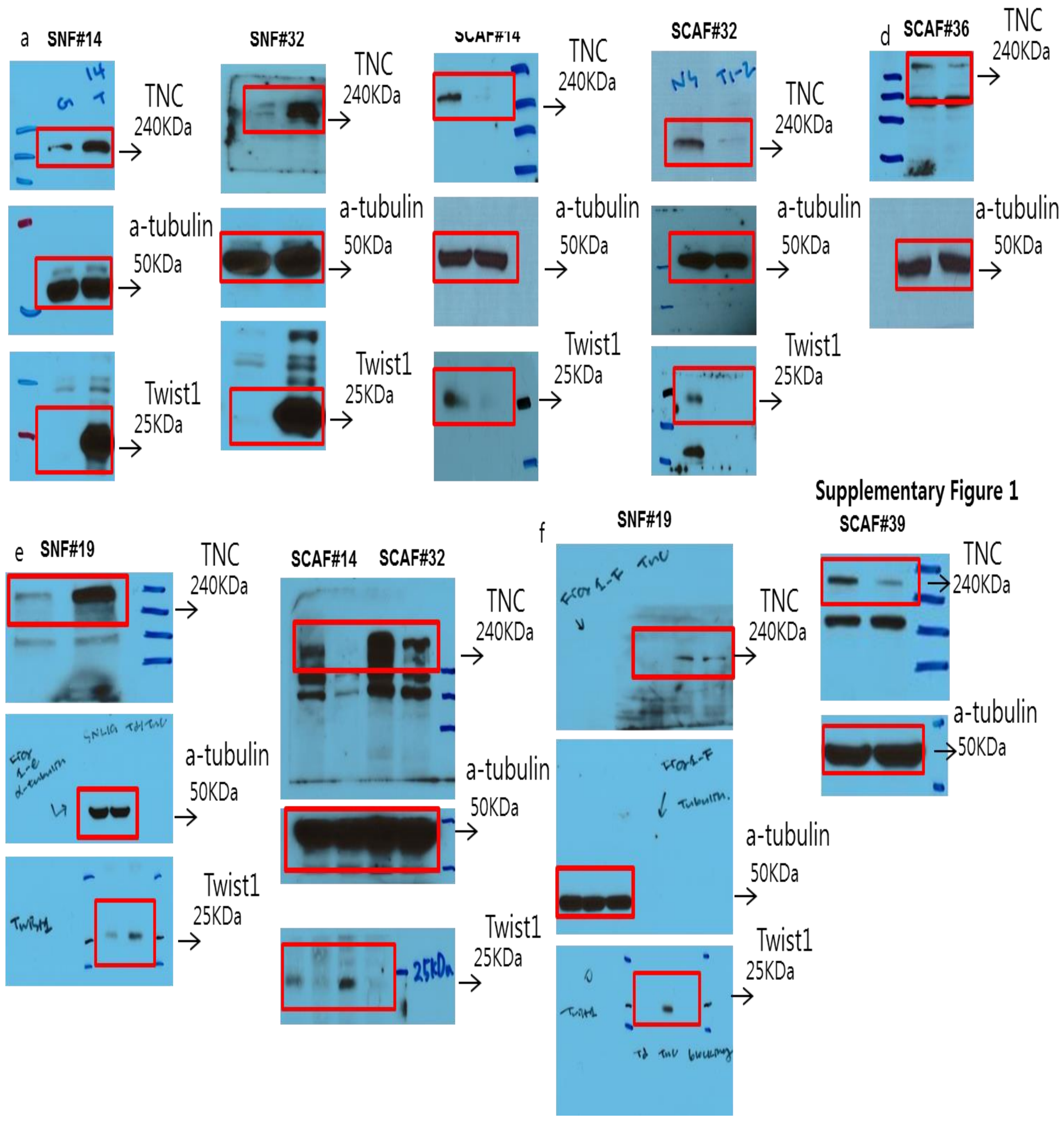


**Supplementary Figure 11**. Uncropped images of western blots with size markers are presented.

Related to Figure 2

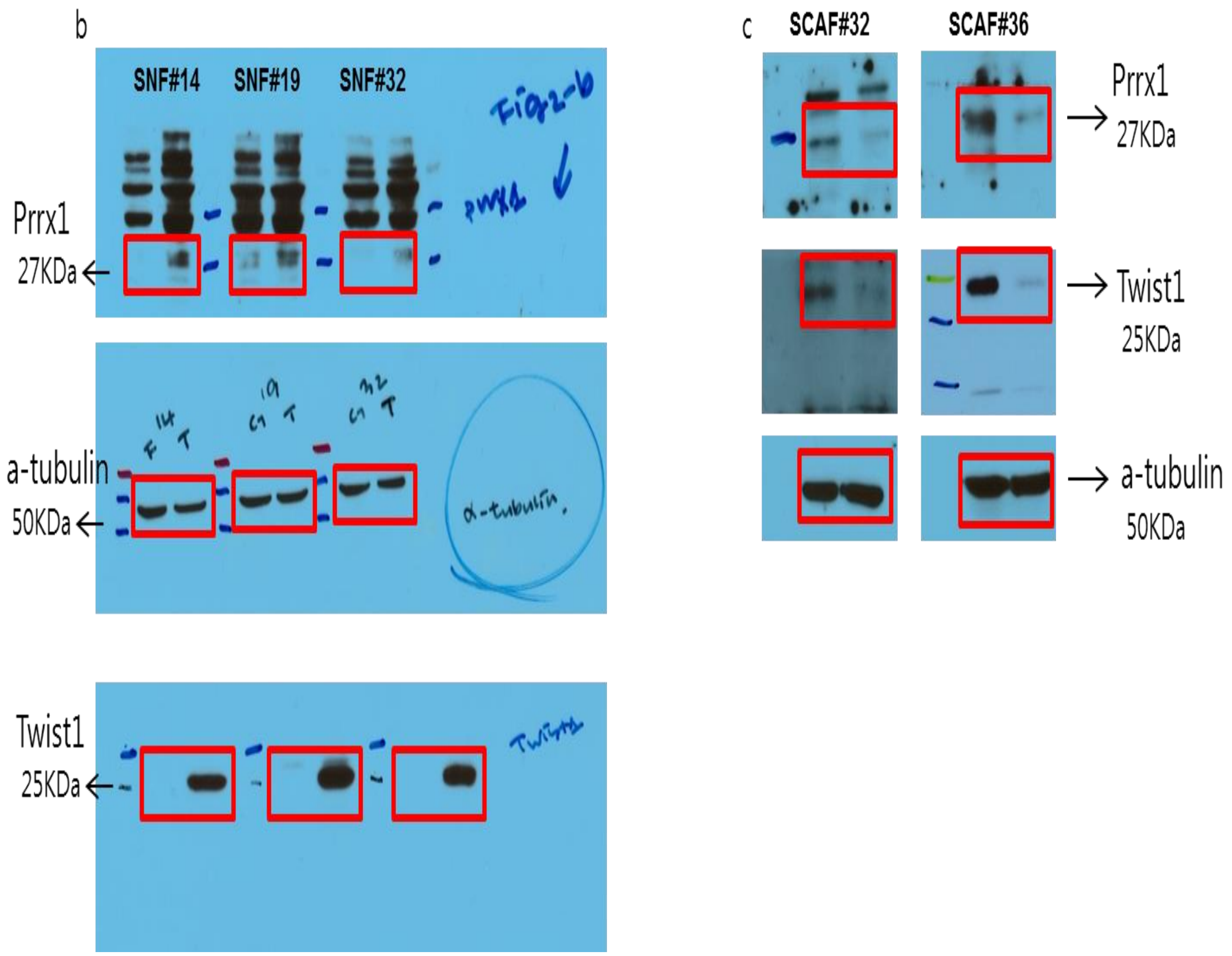


**Supplementary Figure 11 (Continued)**. Uncropped images of western blots with size markers are presented.

Related to Figure 3

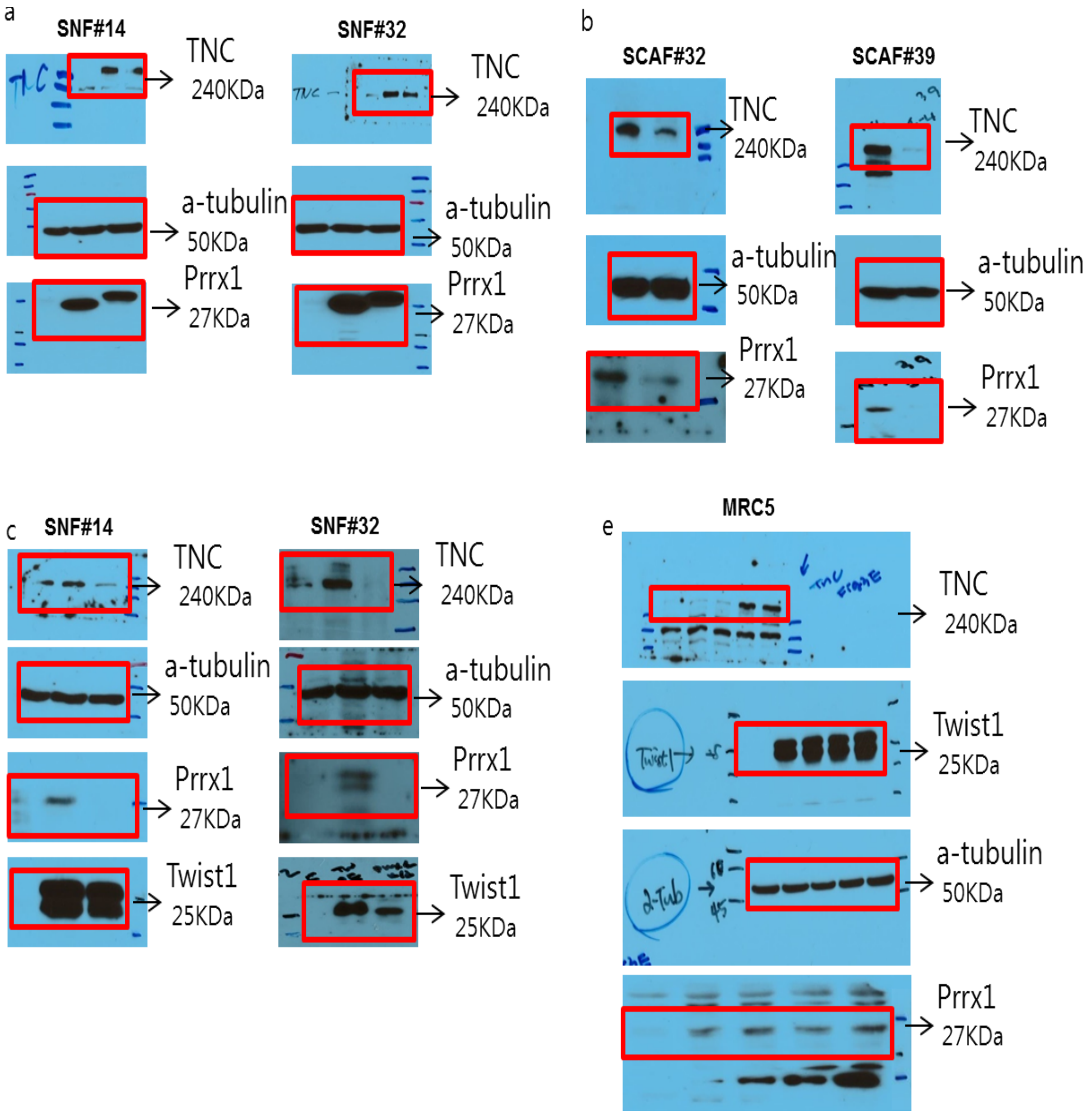


**Supplementary Figure 11 (Continued)**. Uncropped images of western blots with size markers are presented.

Related to Figure 5 and supplementary Figure 5

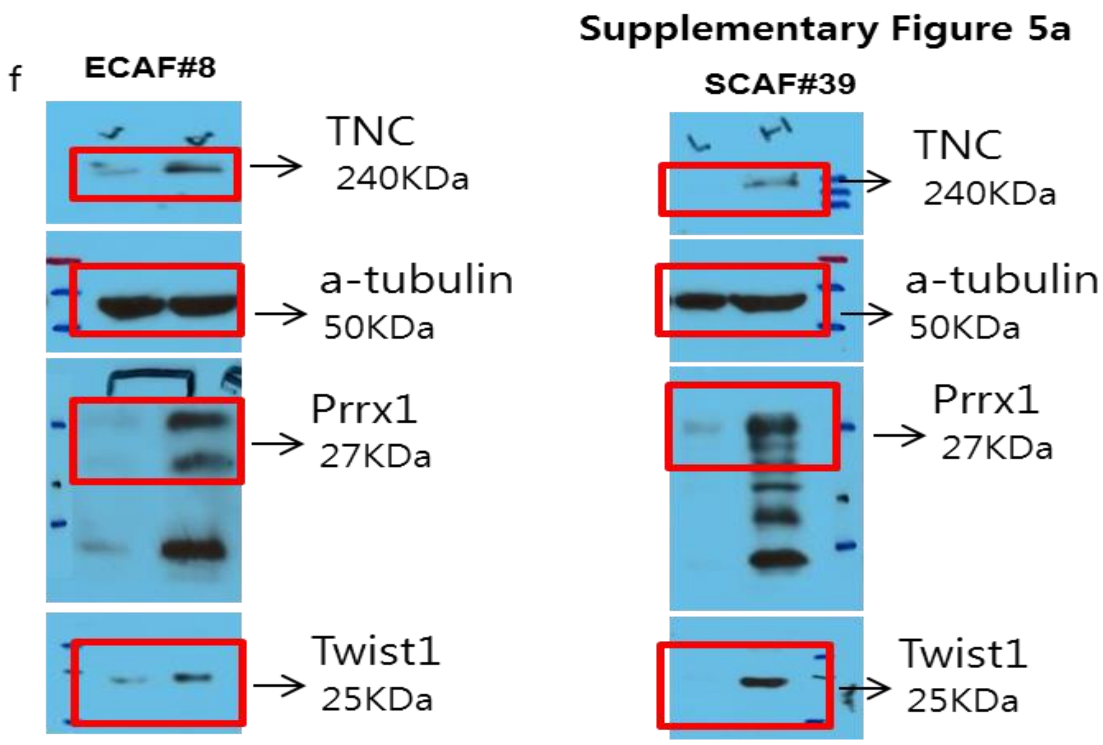


**Supplementary Figure 11 (Continued)**. Uncropped images of western blots with size markers are presented.

Related to Figure7

b

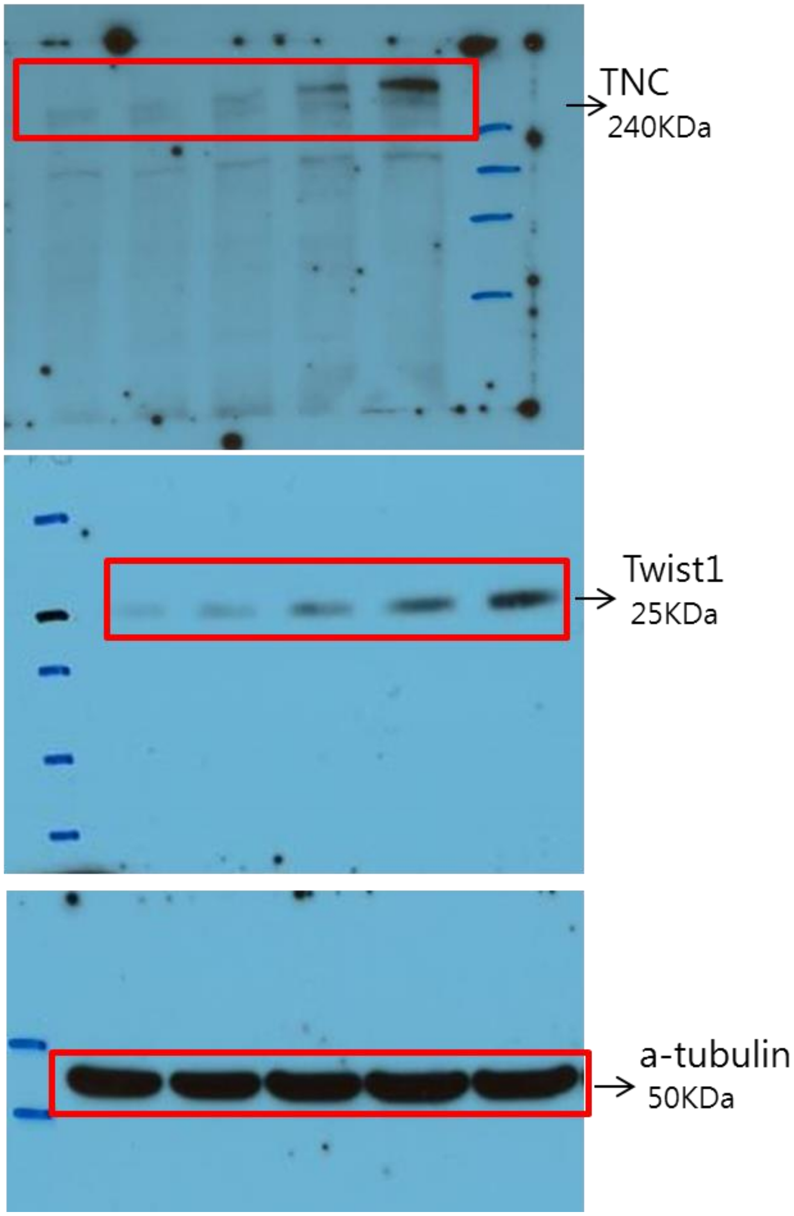


**Supplementary Figure 11 (Continued)**. Uncropped images of western blots with size markers are presented.

**Supplementary Table1**

**Antibodies**

| | | |
|---|---|---|
| Twist1 | Abcam, ab50887 | 1:1000(WB),1:200(IHC/IF), 1ug/ml(ChIP) |
| a-Tubulin | Santacruz, TU-02 | 1:3000(WB) |
| Tn-c | Abcam, ab108930 | 1:500(IHC/IF) |
| Tn-c | GENETEX, GTX62552 | 1:1000(WB) |
| Prrx1 | sigma-aldrich, HPA051084 | 1:200(IHC/IF) |
| Prrx1 | Origene, TA803116 | 1:1000(WB) |
| 2nd goat anti-mouse-HRP | Santacruz, sc 2005 | 1 : 10000(W/B) |
| 2nd goat anti-rabbit-HRP | Santacruz, sc 2004 | 1 : 10000(W/B) |
| Normal mouse IgG | Santacruz, sc2025 | 1ug/ml(ChIP) |

## Supplementary Table 2

| For ChIP | | | |
|---|---|---|---|
| TNC primer 1 | F: AGCAAAGTATGCCGTCGTCT | Prrx1 primer 1 | F: TGCCTTTTGAAGTGCCA |
| | R: AGGCAGAAGATGGGGAAGAG | | R: TCTTGCTGGGCTCCTTT |
| TNC primer2 | F: ATCTGGACGACAGGCAGACT | N.C region | F: TGGATGGATGGATGGAT |
| | R: AGGCAGTGAAGGGACTAGCA | | R: GTAGGTGTCTTCTGTGCATT |
| TNC primer 3 | F: GACATCTCAGGGCCGAAAAC | P.C PDGFRa | F : GCACTATCCCTGGAGTCAGC |
| | R: CGGCGCTTGGAGTGATAGAA | | R : CACCCAGTTGTCCTTCTGGT |
| TNC primer 4 | F: CCAAGAAGGGCCAGTCTACGT | | |
| | R: GCCCCACTTTCTTGATCATTG | | |

| For RT-PCR | Sequences |
|---|---|
| Twist1 | F : TCCAGAGAAGGAGAAAATGGA |
| | R : CCCACGCCCTGTTTCTTTGA |
| Prrx1 | F: CAGGCGGATGAGAACGTGG |
| | R: AAAAGCATCAGGATAGTGTGTCC |
| Tn-C | F: TCCCAGTGTTCGGTGGATCT |
| | R: TTGATGCGATGTGTGAAGACA |
| GAPDH | F: GCACCGTCAAGGCTGAGAA |
| | R: AGCATCGCCCCACTTGATT |

| For Cloning | |
|---|---|
| Twist1 lentiviral cloning (BamHI/Xhc | F: ATTGGATCCATGATGCAGGACGTGTCCAGC |
| | R: ATTCTCGAGCTAGTGGGACGCGGACATG |
| Prrx1a lentiviral cloning (BamHI/Xhc | F: ATTGGATCCATGACCTCCAGCTACGGG |
| | R: ATTCTCGAGTCAGTTGACTGTTGGCACCTG |
| Prrx1b lentiviral cloning (BamHI/Xhc | F: ATTGGATCCATGACCTCCAGCTACGGG |
| | R: ATTCTCGAGTCAGTTGACTGTTGGCACCTG |
| TNC lentiviral cloning (BglⅡ/salI) | F: ATTAGATCTATGGGGGCCATGACT |
| | R: ATTGTCGACTTATGCCCGTTTGCG |
| shTwist1-2 | CCGGCCTGAGCAACAGCGAGGAAGACTCGAG TCTTCCTCGCTGTTGCTCAGGTTTTTG |
| shNS | CCGGCAACAAGATGAAGAGCACCAACTCGAG TTGGTGCTCTTCATCTTGTTGTTTTTG |
| shPrrx1-5 | CCGGGCAGGCTTTGGAGCGTGTCTTCTCGAGAAGAC ACGCTCCAAAGCCTGCTTTTTG |
| shPrrx1-6 | CCGGAGCAGCGAAGGAATAGGACAACTCGAGTTGTCCTA TTCCTTCGCTGCTTTTTTG |
| shTenascin-C | CCGGGGAGTACTTTATCCGTGTATTCTCGAGAATACACG GATAAAGTACTCCTTTTTG |

| For luciferase & mutant assay | |
|---|---|
| pGL3.Prrx1 (650bp) | F: ATTATCGATTGAAACTTTAGTCACTCCTGAGAATC |
| | R: ATTACGCGTGCTCGAATCAACACCAAA |
| pGL3.Prrx1(850bp) | F: ATTGGTACCGCTTCTTGATCCAACTGAGA |
| | R: ATTACGCGTGCTCGAATCAACACCAAA |
| pGL3.Prrx1(1.2kb) | F: ATTATCGATTCAGTTTGTCTTTTCTCCCCGT |
| | R: ATTACGCGTGCTCGAATCAACACCAAA |
| pGL3.Prrx1(3.2kb) | F: ATTGGTACCCCACACCTTTGTCACGAC |
| | R: ATTACGCGTGCTCGAATCAACACCAAA |
| E-BOX 1 mutant | F: CTGCTCTCTAATAGGTCTGCAAGACATC |
| | R: GATGTCTTGCAGACCTATTAGAGAGCAG |
| E-BOX 2 mutant | F: CCAGAGAGCTTCTTGATCTCACTGAGAAGGAAAAAGG |
| | R: CCTTTTTCCTTCTCAGTGAGATCAAGAAGCTCTCTGG |

## Supplementary Table3

| Circos plot GSEA | | | | |
|---|---|---|---|---|
| Cancer Type | Pathway | NES | p-val | FDR-qval |
| Colon adenocarcinoma | Angiogenesis | 2.182591 | 0 | 0 |
| | Epithelial mesenchymal transition | 2.117569 | 0 | 2.57E-04 |
| | Extracellular matrix organization | 2.098079 | 0 | 0.002854 |
| | Apoptosis | 1.931974 | 0 | 0.005136 |
| | P53 | 1.961539 | 0 | 0.002719 |
| Stomach cancer | Angiogenesis | 1.953737 | 0.006148 | 0.007915 |
| | Epithelial mesenchymal transition | 2.302105 | 0 | 0 |
| | Extracellular matrix organization | 2.118408 | 0.00189 | 0.001771 |
| | Apoptosis | 1.796946 | 0.006356 | 0.021135 |
| | P53 | 1.999769 | 0.001942 | 0.003431 |
| Lung squamous cell carcinoma | Angiogenesis | 1.870668 | 0 | 0.029496 |
| | Epithelial mesenchymal transition | 2.350822 | 0 | 0 |
| | Extracellular matrix organization | 2.250397 | 0 | 5.29E-04 |
| Esophageal squamous cell carcinoma | Angiogenesis | 2.044959 | 0 | 0.006631 |
| | Epithelial mesenchymal transition | 2.200889 | 0 | 0 |
| | Extracellular matrix organization | 2.084637 | 0 | 0.014955 |
| | P53 | 1.799126 | 0.004032 | 0.028508 |
| Head and Neck cancer | Angiogenesis | 2.01363 | 0 | 0.003906 |
| | Epithelial mesenchymal transition | 2.170273 | 0 | 0 |
| | Extracellular matrix organization | 2.424593 | 0 | 0 |
| Breast cancer | Angiogenesis | 2.024872 | 0 | 9.80E-04 |
| | Epithelial mesenchymal transition | 2.178462 | 0 | 0 |
| | Extracellular matrix organization | 2.048585 | 0 | 0.002303 |
| | Apoptosis | 1.994614 | 0 | 0.00133 |
| | P53 | 1.985845 | 0 | 9.43E-04 |
| Endometrial cancer | Angiogenesis | 2.107126 | 0 | 0.002851 |
| | Epithelial mesenchymal transition | 2.432389 | 0 | 0 |
| | Extracellular matrix organization | 2.27104 | 0 | 0.003206 |
| | P53 | 1.727642 | 0 | 0.021373 |
| Melanoma | Angiogenesis | 1.938769 | 0 | 0.013485 |
| | Epithelial mesenchymal transition | 2.234258 | 0 | 0 |
| | Extracellular matrix organization | 2.087661 | 0 | 0.045073 |
| Pancreatic cancer | Angiogenesis | 1.875206 | 0.00216 | 0.007089 |
| | Epithelial mesenchymal transition | 2.009837 | 0 | 0.004285 |
| | Extracellular matrix organization | 1.983033 | 0 | 0.004813 |
| | Apoptosis | 1.902175 | 0.001972 | 0.004978 |
| | P53 | 1.952217 | 0 | 0.001351 |
| Prostate cancer | Epithelial mesenchymal transition | 1.71524 | 0 | 0.040288 |
| | Extracellular matrix organization | 1.904468 | 0.002049 | 0.032039 |
| | Apoptosis | 1.705068 | 0.005906 | 0.037308 |
| | P53 | 1.920413 | 0 | 0.005891 |
| Renal cell carcinoma | Angiogenesis | 2.218367 | 0 | 0 |
| | Epithelial mesenchymal transition | 2.218367 | 0 | 0 |
| | Extracellular matrix organization | 2.293175 | 0 | 0 |
| | Apoptosis | 1.889164 | 0.00188 | 0.014866 |
| Rectal cancer | Angiogenesis | 1.96879 | 0 | 0.005118 |
| | Epithelial mesenchymal transition | 2.320097 | 0 | 0 |
| | Apoptosis | 1.924187 | 0.00202 | 0.007461 |
| | P53 | 2.131963 | 0 | 4.81E-04 |
| Uterine Cervical cancer | Angiogenesis | 1.831947 | 0.007273 | 0.030457 |
| | Epithelial mesenchymal transition | 2.140448 | 0 | 0 |
| | Extracellular matrix organization | 2.072113 | 0 | 0.009493 |

## Supplementary Table 4

**Parameter estimates of the mathematical model.**

| Symbol | Unit | Value |
|---|---|---|
| $K_{in}$ | $ng^{-1}\cdot hr^{-1}$ | 6.053E+01 |
| $K_{Tw}^{+}$ | $hr^{-1}$ | 9.348E+00 |
| $V_{TNC\rightarrow Tw}$ | $hr^{-1}$ | 2.997E+01 |
| $K_{m1}$ | - | 3.201E+00 |
| $K_{Tw}^{-}$ | $hr^{-1}$ | 9.349E+00 |
| $n_1$ | - | 9.000E+00 |
| $K_{\mathrm{Pr}}^{+}$ | $hr^{-1}$ | 1.503E+01 |
| $V_{Tw\rightarrow \mathrm{Pr}}$ | $hr^{-1}$ | 1.400E+01 |
| $K_{m2}$ | - | 6.597 E+00 |
| $K_{\mathrm{Pr}}^{-}$ | $hr^{-1}$ | 1.503E+01 |
| $n_2$ | - | 5.000E+00 |
| $K_{TNC}^{+}$ | $hr^{-1}$ | 2.400E-03 |
| $V_{\mathrm{Pr}\rightarrow TNC}$ | $hr^{-1}$ | 1.507E+01 |
| $K_{m3}$ | - | 1.256E+01 |

| | | |
|---|---|---|
| $K_{TNC}^{-}$ | $hr^{-1}$ | 1.000E-02 |
| $n_3$ | - | 3.000E+00 |

## Supplementary Note 1

**Mathematical model of the Twist1/Prrx1/TNC PFL**

We developed a mathematical model of the Twist1/Prrx1/TNC PFL. For this model, we did not attempt to describe the exact *in silico* replica of all biochemical species and their interactions. Instead, we constructed a minimal essential model of the PFL by including only the three major components of it. From our own experimental observations, we confirmed the functional existence of a positive feedback loop formed by these three components: (i) Up-regulation of Twist1 in normal fibroblasts induced both the expression and stabilization of Prrx1; (ii) Prrx1 enhanced both mRNA and protein levels of TNC in normal fibroblasts; (iii) Exogeneous TNC increased Twist1 expression in normal fibroblast. Therefore, we described the regulatory mechanism among these three components based on Michaelis-Menten kinetics. The mathematical description of Twist1 ([Tw]), Prrx1 ([Pr]), and TNC ([TNC]) is summarized in equations (1) to (3) as follows:

$$\frac{d[\mathrm{Tw}]}{dt} = K_{in}[\mathrm{input}] + K_{Tw}^{+} + V_{TNC\to Tw}\frac{[\mathrm{TNC}]^{n1}}{K_{m1}^{n1}+[\mathrm{TNC}]^{n1}} - K_{Tw}^{-}[\mathrm{Tw}] \quad (1)$$

$$\frac{d[\mathrm{Pr}]}{dt} = K_{\mathrm{Pr}}^{+} + V_{Tw\to \mathrm{Pr}}\frac{[\mathrm{Tw}]^{n2}}{K_{m2}^{n2}+[\mathrm{Tw}]^{n2}} - K_{\mathrm{Pr}}^{-}[\mathrm{Pr}] \quad (2)$$

$$\frac{d[\mathrm{TNC}]}{dt} = K_{TNC}^{+} + V_{\mathrm{Pr}\to TNC}\frac{[\mathrm{Pr}]^{n3}}{K_{m3}^{n3}+[\mathrm{Pr}]^{n3}} - K_{TNC}^{-}[\mathrm{TNC}] \quad (3)$$

In equation (1), $K_{in}$ represents the rate constant of the Twist1 production promoted by the doxycycline input. We assumed that the exogenous doxycycline follows the hyperbolic tangent function as follows: $[input] = [doxy]\tanh(t/td)$ where $td$ =20 hours. $K^{+}_{Tw/Pr/TNC}$ and $K^{-}_{Tw/Pr/TNC}$ represents the basal production and degradation rate constants for the three genes. In the equation (1), $V_{TNC\text{->}Tw}$, $K_{m1}$ and $n_1$ represent the maximal production rate, the Michaelis-Menten constant, and the Hill coefficient for activation of Twist1, respectively.

Similarly, $V_{Tw\text{->}Pr}$, $K_{m2}$ and $n_2$ are for Prrx1; $V_{Pr\text{->}TNC}$, $K_{m3}$ and $n_3$ are for TNC. To estimate appropriate parameter values that can reproduce the fold change of the three genes as observed in our experiments, the levels of state variables were normalized by the basal levels of them such that all the state variables have the initial value of '1' at t=0. To investigate the underlying mechanism of persistent activation of PFL upon injection of high TNC, we modified the equation (1) such that the TNC injection induces the expression of Twist1, as follows:

$$\frac{d[\mathrm{Tw}]}{dt} = K_{in}\frac{[\mathrm{input}]^{n4}}{K_{m4}^{n4}+[\mathrm{input}]^{n4}} + K_{Tw}^{+} + V_{TNC\to Tw}\frac{[\mathrm{TNC}]^{n1}}{K_{m1}^{n1}+[\mathrm{TNC}]^{n1}} - K_{Tw}^{-}[\mathrm{Tw}].$$

Using this modified model, we estimated again the parameter values such that the level of Twist1 and TNC is in accord with the experimental observation along with the increase of recombinant TNC concentration (Figure 7b).

**Parameter estimation**

Our model consists of three state variables and 16 kinetic parameters. The kinetic parameter values were estimated based on our own time course measurements by using the genetic algorithm (GA) implemented in Matlab Toolbox™. Parameter estimation was carried out based on maximum likelihood estimation, starting from some initial guess to find the optimal parameter estimates that minimize the difference between experimental data and simulated data obtained from the mathematical model. The cost function ( $J$ ) for parameter estimation is defined by $J(\boldsymbol{p}) = \sum_{j=1}^{M}\sum_{i=1}^{N}(\bar{y}_{j,i} - y_j(t_i, \boldsymbol{p}))^2$ , where $M$ is the number of given experimental data and $N$ is the number of time points of each experimental data. The output of the mathematical model $y_j(t_i, \boldsymbol{p})$ is the value of the solution $y_j$ of the ordinary differential equation (ODE) at $t_i$ with parameter set $\boldsymbol{p}$ and $\bar{y}_{j,i}$ is the mean of given repeated measurements of signaling molecules corresponding to $y_j$ at $t_i$ . All the parameter estimates are summarized in Supplementary Table4.
